# MicroRNA-Based Therapy in Animal Models of Selected Gastrointestinal Cancers

**DOI:** 10.3389/fphar.2016.00329

**Published:** 2016-09-27

**Authors:** Jana Merhautova, Regina Demlova, Ondrej Slaby

**Affiliations:** ^1^Molecular Oncology II – Solid Cancer, Central European Institute of Technology, Masaryk UniversityBrno, Czech Republic; ^2^Department of Pharmacology, Faculty of Medicine, Masaryk UniversityBrno, Czech Republic; ^3^Masaryk Memorial Cancer InstituteBrno, Czech Republic

**Keywords:** microRNA, gastric cancer, pancreatic cancer, gallbladder cancer, colorectal cancer, animal model, mice, preclinical testing

## Abstract

Gastrointestinal cancer accounts for the 20 most frequent cancer diseases worldwide and there is a constant urge to bring new therapeutics with new mechanism of action into the clinical practice. Quantity of *in vitro* and *in vivo* evidences indicate, that exogenous change in pathologically imbalanced microRNAs (miRNAs) is capable of transforming the cancer cell phenotype. This review analyzed preclinical miRNA-based therapy attempts in animal models of gastric, pancreatic, gallbladder, and colorectal cancer. From more than 400 original articles, 26 was found to assess the effect of miRNA mimics, precursors, expression vectors, or inhibitors administered locally or systemically being an approach with relatively high translational potential. We have focused on mapping available information on animal model used (animal strain, cell line, xenograft method), pharmacological aspects (oligonucleotide chemistry, delivery system, posology, route of administration) and toxicology assessments. We also summarize findings in the field pharmacokinetics and toxicity of miRNA-based therapy.

## Introduction

**Figure 1:**
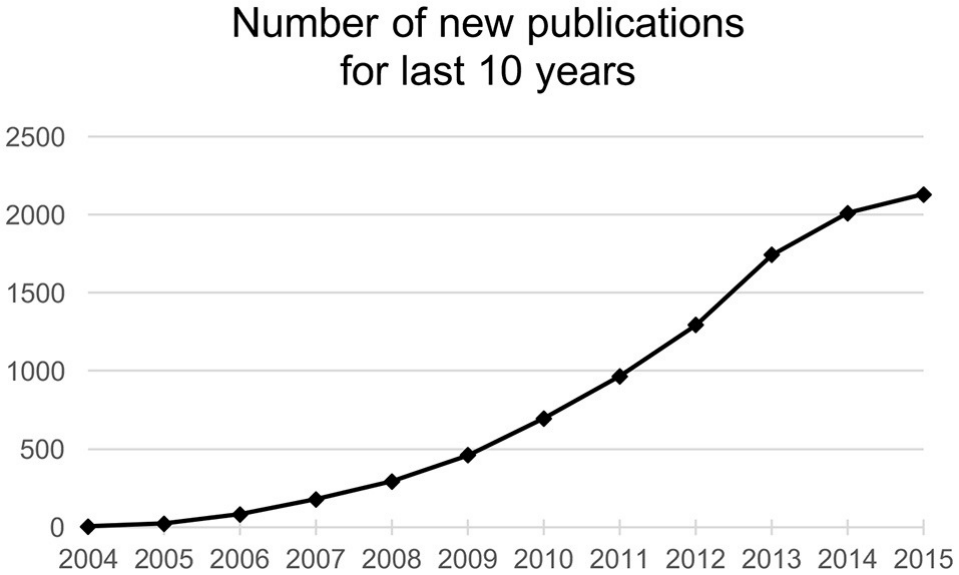
**Number of new publications found by the term “miRNA AND cancer” in SCOPUS database**.

Research in the field of non-coding nucleic acids has advanced extensively in the last 15 years. It is now well known, that dysregulation of miRNAs, powerful regulators of gene expression, is associated with many diseases. MiRNAs are investigated thoroughly in cancer biology and oncology and the number of published articles is growing (Figure 1). Last 10 years brought us an immense amount of information about the roles of miRNAs in cancer cell pathophysiology. All described hallmarks of cancer (Hanahan and Weinberg, 2011) are in relation with some miRNA imbalance (Ruan et al., 2009). Attempts to therapeutically interfere with miRNAs levels in pathologic cells are moving forward to preclinical and clinical phases of new therapies development. Although there are severe limitations and barriers facing miRNA-based therapy, more and more studies are performed with auspicious results.

The purpose of this review is to analyze preclinical studies carried out on animal models of selected gastrointestinal cancer (gastric, pancreatic, gallbladder, and colorectal). We have focused primarily on pharmacological aspects of miRNA-based therapy with the emphasis on delivery systems, and also on the type of animal model, and on toxicity assessments. Eventually, we summarize important findings in the field pharmacokinetics and toxicity of miRNA-based therapy to make the picture comprehensive.

### The biogenesis of endogenous miRNAs

MiRNAs are endogenous small (~22 nt) single-stranded non-coding RNAs. Their main role in the cell lies in post-transcriptional attenuation of mRNA translation. The biosynthesis of miRNAs begins in the nucleus. Long double-stranded transcripts (pri-miRNAs) are formed by RNA-polymerases II and III. Pri-miRNAs are cleaved by the ribonuclease Drosha and DGCR8 protein to form pre-miRNAs, double-stranded chains ~70 nt long. Pre-miRNAs are then transported from the nucleus to the cytoplasm via Exportin-5 protein. In the cytoplasm, mature miRNAs are created through the interaction with endonuclease Dicer and TRBP protein. Double-stranded formation is rearranged, the guide strand forms a complex with Argonaut and other proteins forming miRISC complex, and plays an active role in the gene expression attenuation. The other strand, called passenger strand, is usually degraded in the cytoplasm, or persists and may exert its own biological activity. For detailed information on miRNA biogenesis see a recent review by Romero-Cordoba et al. (2014).

There are also number of proofs of mature miRNAs' presence and activities in the nucleus (Hwang et al., 2007; Park et al., 2010; Jeffries et al., 2011; Li et al., 2013). It seems that these miRNAs could transfer from cytoplasm to nucleus and nucleolus via Exportin-1 and Importin-8 (Li et al., 2013; Wei et al., 2014) and influence expression of other miRNAs, or of its own (Tang et al., 2012; Zisoulis et al., 2012; Wei et al., 2014).

### Mechanism of action

**Figure 2:**
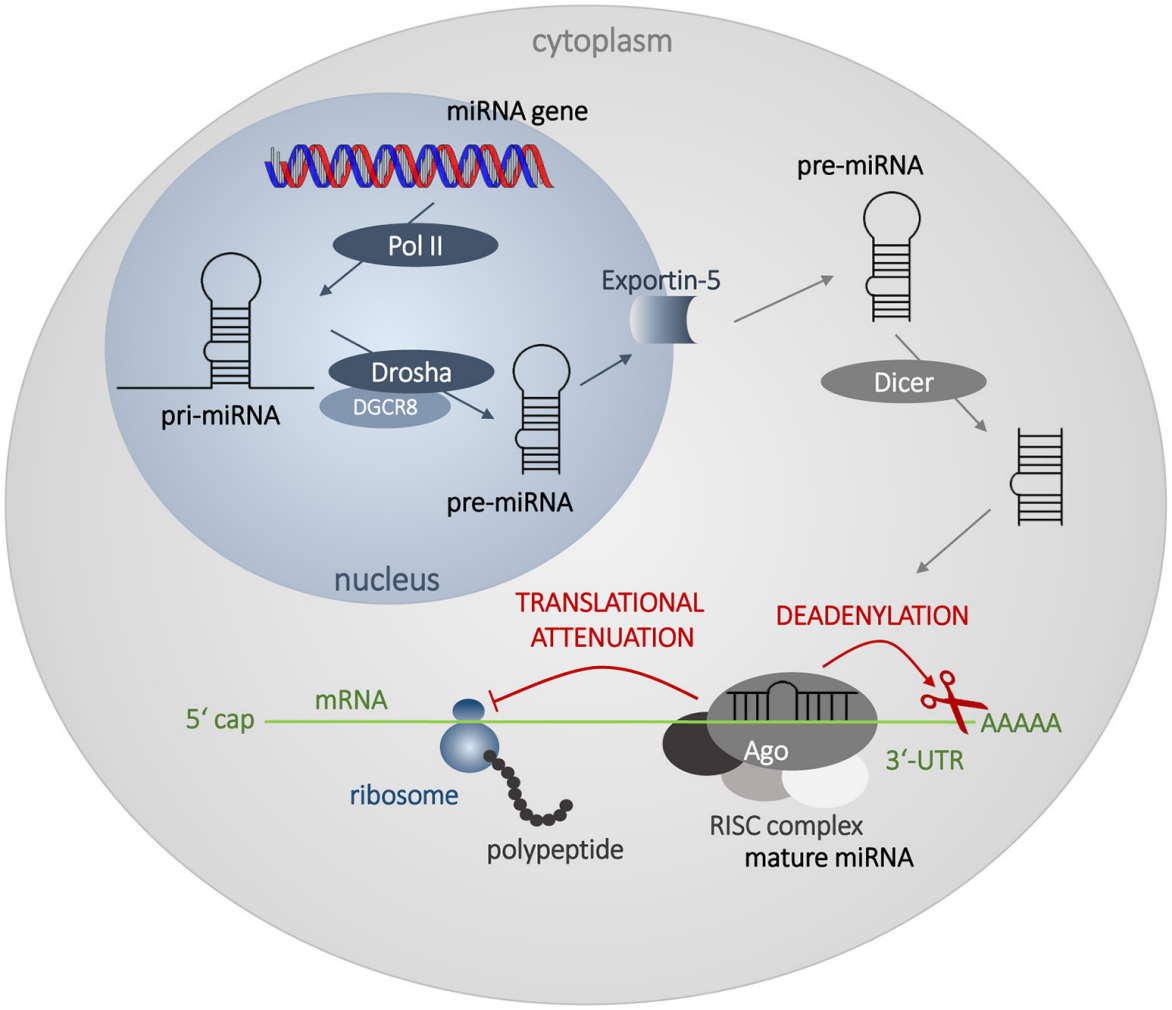
**Biosynthesis and mechanism of action of miRNAs**. The biosynthesis begins in the nucleus by transcription of miRNA genes by RNA polymerase II (Pol II). Long transcripts, pri-miRNAs, are cleaved by Drosha and DGCR8 protein creating pre-miRNA with hairpin structure. Exportin 5 transfers pre-miRNA into the cytoplasm, where it is processed by Dicer into miRNA duplex. Mature single-strand miRNA forms RISC complex with Argonaut (Ago) and other proteins and attenuates mRNA translation and leads to the destabilization of mRNA by deadenylation.

MiRNAs bind mainly to the 3′-untranslated region of mRNA (3′-UTR), although there are several evidences that miRNAs could bind to the 5′-UTR, or to the coding sequence itself (Ott et al., 2011; Gu et al., 2014). In the case of imperfect matching, the duplex mRNA:miRNA is not translated, or it is translated incompletely and the polypeptide chain is subsequently degraded. Binding of miRNA to mRNA target also activates deadenylation of 3′-poly(A) end of mRNA through deadenylases, which is a first step of mRNA destabilization and later degradation by 3′- and 5′-exonucleases (Figure 2). Perfect matching leads to direct cleavage of the target mRNA. Imperfect matching is more common in animal cells, while perfect matching is typical for plant cells (Axtell et al., 2011). The binding specificity is ensured by the seed sequence of miRNA, which contains 6–8 nt and which is very often conservative through the species (Hogg and Harries, 2014). One miRNA can regulate many different genes, and more than 50% of all genes are suggested to be regulated by miRNAs. Thus, miRNA network affect most of cellular processes from the basic metabolic maintenance, through differentiation, cell division and proliferation, to the death (Calin and Croce, 2006; Esquela-Kerscher and Slack, 2006; Garzon et al., 2006; Zhang et al., 2013).

In cancer tissues, a lot of changes in miRNA levels could be found. MiRNAs decreased in cancer cells are termed tumor suppressors and reversely, oncogenic miRNAs are those abundant in cancer tissue. There have already been signs of miRNAs that function both as tumor suppressors, and oncogenes depending on the cell type and state (context-dependent miRNAs) (Esquela-Kerscher and Slack, 2006; Kasinski and Slack, 2011).

**Figure 3:**
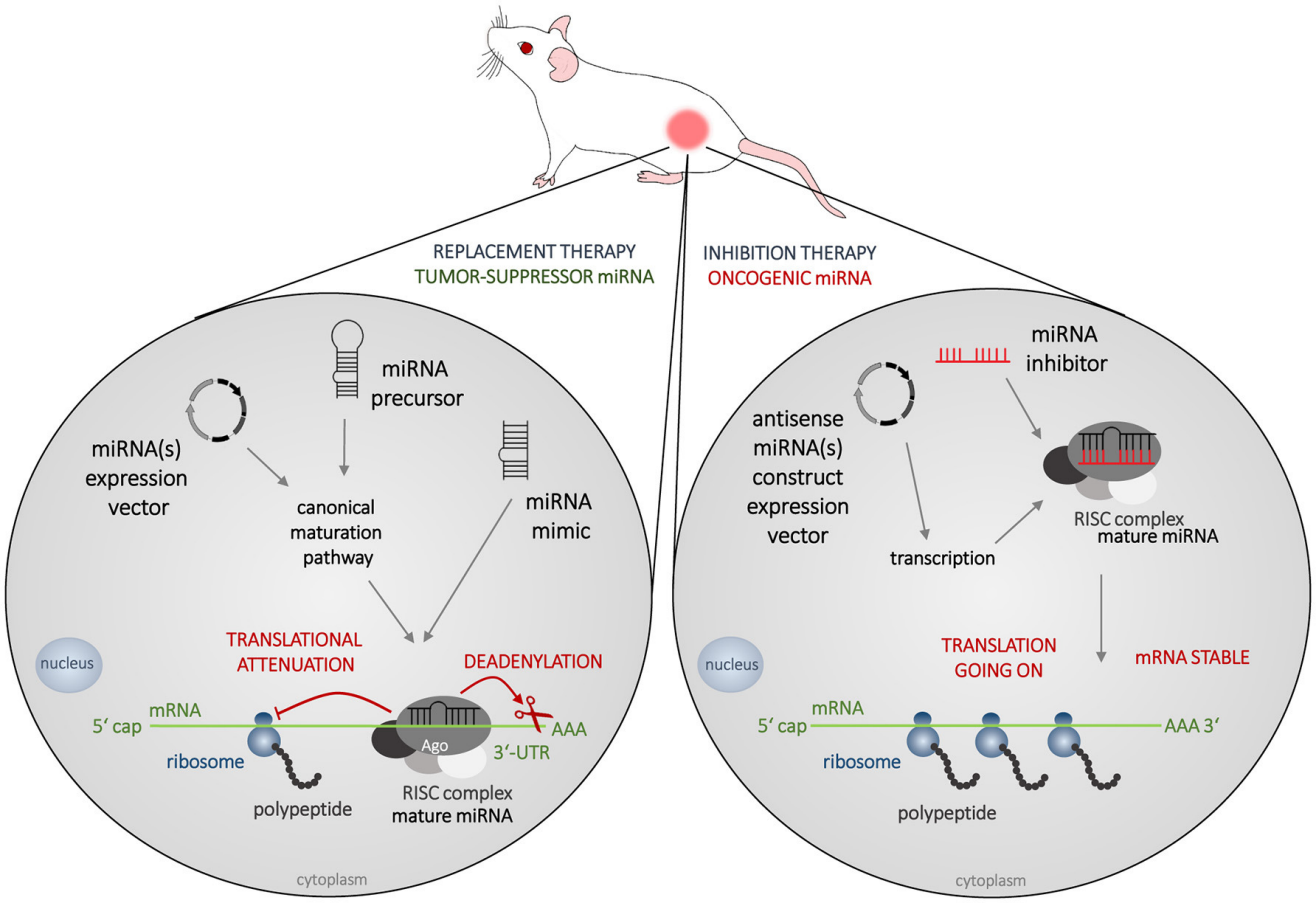
**Strategies in miRNA-based therapy**. The most frequently used animal model of cancer is immunodeficient mouse bearing a subcutaneous tumor created from cells of human origin. In miRNA-based therapy, two concepts are adopted nowadays, which is the replacement therapy (left) and inhibition therapy (right). Tumor suppressors MiRNAs are decreased in cancer cells and to increase their levels mature miRNAs, miRNA-mimics, precursors, or expression vectors are administered. Oncogenic miRNAs are abundant in cancer tissue and to silence their effects, various types of miRNA inhibitors could be administered.

To reverse the pathologic imbalance of miRNAs mature miRNAs, miRNA-mimics, precursors, or expression vectors are administered to increase the level of a specific tumor-suppressor miRNA, and miRNA inhibitors are administered to decrease the level of oncogenic miRNA (Figure 3). Promising results of *in vitro* studies are nowadays being verified on animal models and first preclinical, or even clinical trials are under way.

## Search strategy

Web of Science database was searched for *in vivo* studies published in the last 5 years (2010–2015) that were focused on colorectal, pancreatic, gallbladder and gastric cancer. Searching formulas *miRNA AND vivo AND colorectal/pancreatic/gallbladder/gastric* in article topic (title, abstract and keywords) was used. The search was finished by the end of February 2016. About 430 articles were found and further analyzed to select the specific experimental design: at first, induction of a tumor by transplantation of human or murine tumor cells, or tumor tissue, then followed by the administration of miRNA mimic, precursor, expression vector, or inhibitor. The bulk of the studies found by the searching formula were excluded because of using different methods, e.g., influencing the expression level of miRNA in cancer cells before transplantation into the animal body, or administration of other substances that affect miRNA levels and processes like natural compounds, siRNAs etc. 26 studies included in this review matched the aforementioned criteria.

Both the articles themselves and the supplemental materials were scrutinized with accent on animal model used (animal strain and gender, xenograft method, cancer cell line, or source), pharmacological aspects (oligonucleotide chemistry, delivery system, posology, route of administration), toxicology assessments (methods and findings), and eventually the experimental therapy effect. Some of the information could not be obtained from articles or supplements, as they lacked e.g., animal gender specification.

## Overview of the selected studies

**Figure 4:**
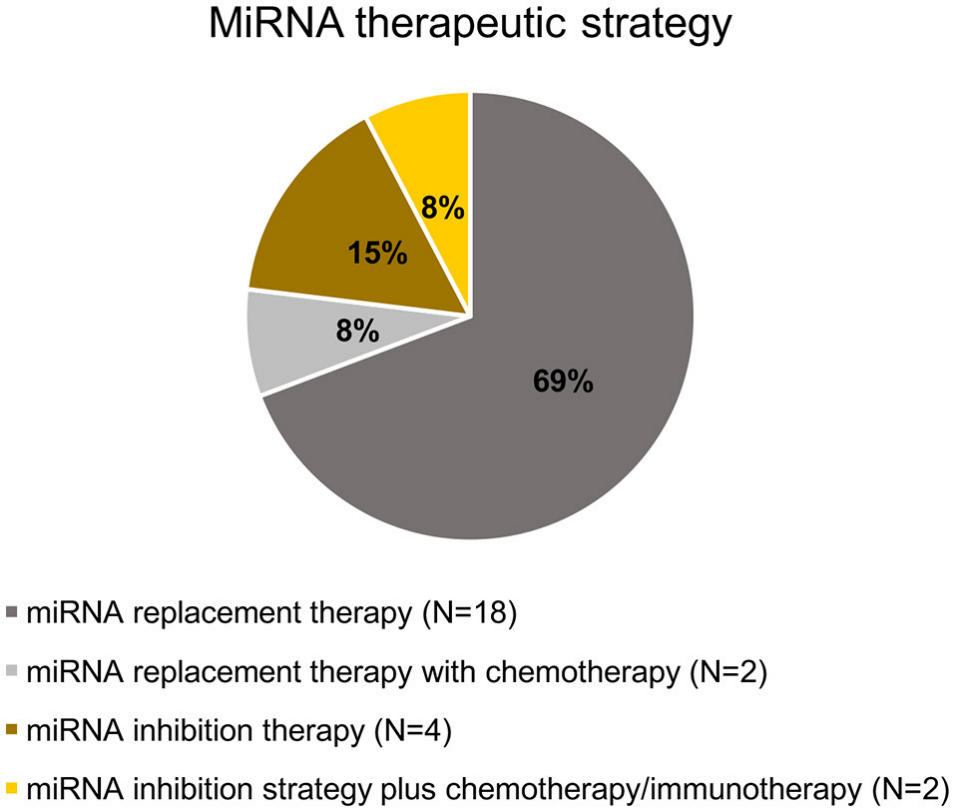
**Pie chart of miRNA therapeutic strategy in the selected studies**.

**Figure 5:**
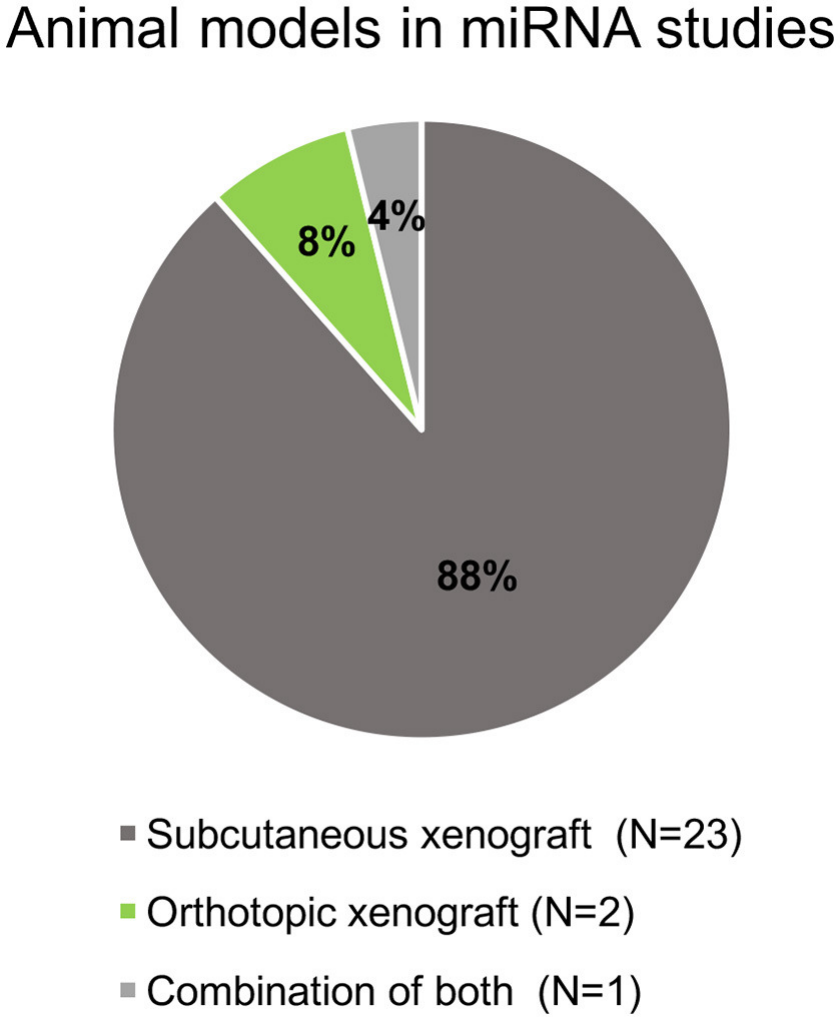
**Pie chart of animal models and xenograft methods in the selected studies**.

**Figure 6:**
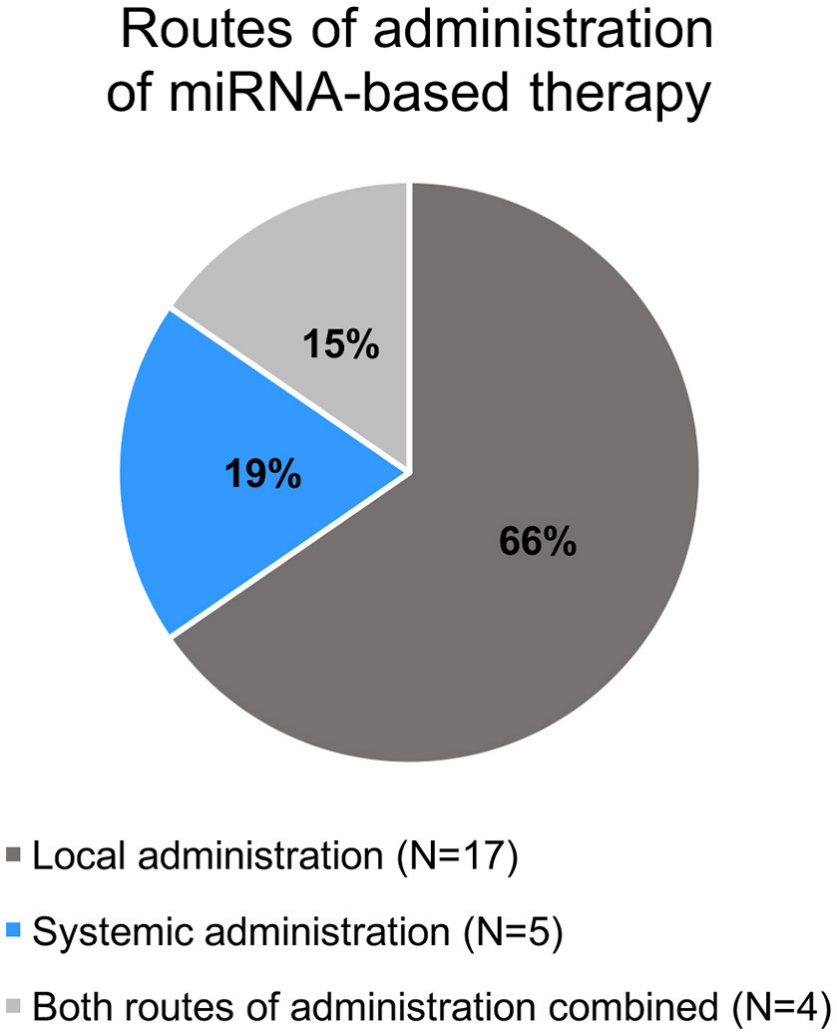
**Pie chart of routes of administration of miRNA-based therapy in the selected studies**.

All selected studies assorted by the organ of cancer cells' origin are summarized in **Tables 2–5**. Visual summary of therapeutic strategy, type of animal model and routes of administration of miRNA-based therapy is demonstrated in Figures 4–6. We have analyzed 26 studies, 20 of them used the miRNA replacement therapy regimen, and others were miRNA inhibitions. Two studies combined miRNA replacement therapy with chemotherapy, two studies combined miRNA inhibition with either chemotherapy, or immunotherapy. Subcutaneous xenograft model was used in 23 cases, orthotopic xenotransplantation was performed in two experiments, and combination of both was done in one study. In 17 studies, miRNA-based therapeutics were administered locally, i.e., injected intratumorally. Five studies involved systemic administration by tail-vein, or intraperitoneal injection, while four studies combined both routes of administration in a separate substudies, or combined systemic administration of e.g., chemotherapy, with local administration of miRNA-based therapy.

MiRNAs studied in the selected articles were both known tumor suppressors, or oncogenes, and also context-dependent miRNAs whose effect varies according to the type of cancer cell. All of them influence the main hallmarks of cancer such as uncontrolled tumor cell proliferation, impaired process of apoptosis, defects in the control of cell cycle, increased migration and invasivity, or tumor angiogenesis (Table 1). Some miRNAs were tested in combination with cytostatic agents (doxorubicin, gemcitabine, or oxaliplatin) to achieve sensitization of chemotherapy-resistant cells and tumors, e.g., by decreasing the expression of efflux proteins such as ABCB1 (P-glycoprotein). The main result of all *in vivo* studies was inhibition of tumor xenograft growth, at least in a transient manner. All results and references could be found in Tables 2–5.

**Table 1:** **Examples of studied miRNAs in association with some of the hallmarks of cancer and other cancer cells attributes (I, inhibition strategy; R, replacement strategy)**.

**Cancer cell attribute**	**Studied miRNA**	**Replacement or inhibition strategy**	**References**
Uncontrolled cell proliferation	miR-21	I	Sicard et al., 2013
	miR-27a	R	Bao et al., 2014
	miR-33a	R	Ibrahim et al., 2011
	miR-145	R	Ibrahim et al., 2011
	miR-218	R	He et al., 2012
	miR-429	R	Sun Y. et al., 2014
Impaired apoptosis	let-7	I	Geng et al., 2011
	miR-20a	I	Chang et al., 2013; Wang et al., 2013
	miR-21	I	Frampton et al., 2011; Sicard et al., 2013
	miR-27a	R	Bao et al., 2014
	miR-145	R	Ibrahim et al., 2011
	miR-4689	R	Hiraki et al., 2015
Dysfunction in cell cycle control	miR-133a	R	Dong et al., 2013
	miR-200a	R	Cong et al., 2013
	miR-218	R	He et al., 2012
	miR-1266	R	Chen et al., 2014
Cell migration and invasivity	miR-27a	R/I	Frampton et al., 2011; Bao et al., 2014
	miR-200a	R	Cong et al., 2013
	miR-429	R	Sun Y. et al., 2014
	miR-1207-5p	R	Chen et al., 2014
Neoangiogenesis	miR-27a	I	Frampton et al., 2011
	miR-27b	R	Ye et al., 2013
Resistance to cytostatic agents	miR-103	R	Zhang et al., 2015
	miR-107	R	Zhang et al., 2015

**Table 2:** ***In vivo* studies in animal models of gastric adenocarcinoma**.

**Xenografted cell line**	**MiRNA(s) of interest**	**Therapeutic strategy**	**Animal model**	**Animal strain (gender)**	**Delivery system and chemical modifications**	**Route of administration**	**Dosage**	**Frequency of administration**	**Results**	**Toxicity, undesired effects**	**References**
SGC-7901	miR-17-5p/20a	IT	SX	BALB/c mice	AntagomiR-17-5p and antagomiR-20a (RiboBio)	Intratumoral inj.	25 μmol	Twice weekly for 2 weeks	Inhibition of tumor growth, increase in the percentage of apoptotic cells in tumor tissue	Not assessed	Wang et al., 2013
SGC-7901	miR-200a	RT	SX	BALB/c-A mice	miRNA-mimics (GenePharma) with Lipofectamine 2000 (Invitrogen)	Intratumoral inj.	10 μl	Twice after 2 days	Inhibition of tumor growth	Not assessed	Cong et al., 2013
SGC-7901	miR-1266/1207-5p	RT	SX	Nude mice (females)	Lentiviral vector (Lv-miR-166/1207-5p, GeneChem Management)	Intratumoral inj.	0.1 ml 10^7^ PFU/ml	Single dose	Inhibition of Tumor growth and tumor cells proliferation	Not assessed	Chen et al., 2014
Doxorubicin resistant SGC-7901/ADR^fluc^	miR-16	RT + chemotherapy	SX	BALB/c mice (females)	miRNA oligonucleotides (GenePharma) bound on PEG-coated Fe_3_O_4_ nanoparticles	Tail-vein inj.	5 mg/kg (1 nmol)	Seven times (days 0, 3, 7, 10, 14, 17, 21 post inocul)	Tumor size reduction, increase of number of apoptotic nuclei, increased sensitivity to doxorubicin	No noticeable damage in histology analysis of heart, liver, spleen and kidney	Sun Z. et al., 2014
					Doxorubicin	Intraperitoneal inj.	2.5 mg/kg	Once a week for 4 weeks (days 0, 7, 14, 21)			
Doxorubicin resistant SGC-7901/ADR	miR-103/107	RT + chemotherapy	SX	BALB/c mice	Cholesterol-conjugated 2′-*O*-methyl-modified agomiR-103/107 (RiboBio)	Intratumoral inj.	1 nmol	Every 4 days for seven times	Delayed tumor growth, reduction in tumor volume, lower proliferative potential, increased sensitivity to doxorubicin	No obvious signs of toxicity such as weight loss over the course of the treatment	Zhang et al., 2015
					Doxorubicin	Intraperitoneal inj.	2 mg/kg	Every other day			

*Ad-wt, wild type adenovirus; ALT, alanine transaminase; AST, aspartate aminotransferase; BUN, blood urea nitrogen; DOTAP, N-[1-(2,3-dioleoyloxy)propyl]-N,N,N-trimethylammonium methyl-sulfate; DSPE-PEG, 1,2-distearoyl-sn-glycero-3-phosphoethanolamine-N-[amino(polyethylene glycol)-2000]; GEM, gemcitabine; incl., including; inj., injection; IT, inhibition therapy; mAb, monoclonal antibody; NOD/SCID, non-obese diabetic/severe combined immunodeficiency; OD, optical density; OX, orthotopic xenograft; PEG, polyethylene glycol; PEI, polyethylenimine; PFU, plaque-forming units; post inocul., post inoculation; RT, replacement therapy; SCID, severe combined immunodeficiency; SX, subcutaneous xenograft; TNF-α, tumor necrosis factor α; VP, viral particles*.

**Table 3:** ***In vivo* study in animal models of gallbladder carcinoma**.

**Xenografted cell line**	**MiRNA(s) of interest**	**Therapeutic strategy**	**Animal model**	**Animal strain (gender)**	**Delivery system and chemical modifications**	**Route of administration**	**Dosage**	**Frequency of administration**	**Results**	**Toxicity, undesired effects**	**References**
GBC-SD initially transfected with miR-20a antagomir (200 nM) for 3 days	miR-20a	IT	SX	Nude mice	AntagomiR-20a	Intratumoral inj.	5 nmol	Twice weekly for 2 weeks	Inhibition of tumor growth	Not assessed	Chang et al., 2013

**Table 4:** ***In vivo* studies in animal models of pancreatic ductal adenocarcinoma**.

**Xenografted cell line**	**miRNA(s) of interest**	**Therapeutic strategy**	**Animal model**	**Animal strain (gender)**	**Delivery system and chemical modifications**	**Route of administration**	**Dosage**	**Frequency of administration**	**Results**	**Toxicity, undesired effects**	**References**
Mia PaCa-2 Lucia F1	miR-21	IT	OX	SCID CB 17 mice	Lentiviral vector producing hairpins antisense to miR-21 [LV(a/miR-21)]	Intratumoral inj.	150 ng	Single dose	Inhibition of tumor growth and proliferation, induction of apoptosis, activation of angiogenesis	No changes in body weight	Sicard et al., 2013
		IT + chemotherapy			Lentiviral vector producing hairpins antisense to miR-21 [LV(a/miR-21)]	Intratumoral inj.	150 ng	Single dose	synergic effect, strong inhibition of tumor growth		
					Gemcitabine	Intraperitoneal inj.	125 mg/kg	Twice weekly for 14 days			
MiaPaCa-2	miR-34a, miR-143/145 cluster	RT	SX OX	CD-1 mice	Liposomal nanoparticles from cationic amphiphile DOTAP and co-lipids (cholesterol, DSPE-PEG-OMe) with plasmide pMSCV-puro expression construct of miR-34a and miR-143/145 (Clontech Laboratories)	Tail-vein inj.	50 μg	Three times per week for 3 weeks	Inhibition of tumor growth, more widespread apoptosis	No histopathology or biochemical evidence of toxicity (incl. hematology, liver and renal function)	Pramanik et al., 2011
Capan-1, Capan-2	miR-219-1-3p	RT	SX	SCID CB17 mice (males)	Plasmide pcDNA6.2-miR-219 with ExGen 500 transfection reagent (Euromedex)	Intratumoral inj.	20 μg	Single dose	Decrease of tumor growth and proliferation	Not assessed	Lahdaoui et al., 2014
PANC-1	miR-34a	RT	SX	BALB/c mice (females)	Cationic polymer from PEI and β-cyclodextrin conjugated with CC9 peptide	Tail-vein inj.	15 μmol (20 μg)	Twice weekly for 2 weeks	Inhibition of tumor growth and decrease in size, induction of cancer cell apoptosis	Not assessed	Hu et al., 2013
PANC-1	miR-217	RT	SX	BALB/c mice (males)	miR-217 expression vector *in vivo*-jetPEI™ (201-50G; Polyplus)	Intratumoral inj.	100 μg	twice	Decrease of tumor growth	Not assessed	Zhao et al., 2010
RWP-1	miR-148a	IT	SX	Athymic nu/nu mice (males)	Engineered oncolytic adenovirus Ad-L5-8miR148aT (insertion of 8 target sites for miR-148a)	Intratumoral inj.	5 × 10^10^ VP/tumor	Single dose	Significant inhibition in tumor growth and reduced tumor weight	Less induction of ALT, AST and bilirubin indicative of attenuated viral toxicity than Ad-wt, hepatotoxicity is transient	Bofill-De Ros et al., 2015
Patient derived CP13						Intratumoral inj.					
Patient derived CP15						Intratumoral inj. Tail-vein inj.					
MIA PaCa-2, PANC-1	miR-21/23a/27a	IT	SX	BALB/c mice (females)	AntimiR-21 with atelocollagen	Intratumoral inj.	12 μmol	Once weekly for 3 weeks followed by 3-week pause and again once weekly for 3 weeks	Suppression of tumor growth, the effect was lost after 3 weeks	No death, loss of body weight, or gross adverse effects occurred in the mice	Frampton et al., 2011
MIA PaCa-2, PANC-1	miR-21/23a/27a	IT	SX	BALB/c mice (females)	AntimiR-21/23a/27a with atelocollagen	Intratumoral inj.	4 μmol for each antimiR		Reduction in tumor volume sustained to the end of the experiment despite a 3-week rest period	No death, loss of body weight, or gross adverse effects occurred in the mice	Frampton et al., 2011
PANC-1, PANC10.05	miR-206	RT	SX	SCID mice	mirVana miR-206 mimics (Ambion) with *in vivo*-jet PEI (Polyplus) in 0.015% collagenase II (Sigma) solution	Intratumoral inj.	10 μg	Three times (day 1, 8, 13)	Increased tumor necrosis, no changes in total tumor burden	Not assessed	Keklikoglou et al., 2015
Gemcitabine-resistant MIA PaCa-2^R^	miR-205	RT	SX	athymic nude mice (males)	Gemcitabine conjugated miR-205 polyplexes from amphiphilic copolymer with PEG	Intratumoral inj.	1 mg/kg (GEM 40 mg/kg)	Three times a week for 2 weeks	Reduction in tumor growth rate and weight, reduction in cell proliferation, increase in apoptosis	No significant change in body weight	Mittal et al., 2014
Capan-2, MiaPaCa-2	miR-29a, 330-5p	RT	SX	SCID CB-17 mice (males)	miRNAs cloned into the pCDNA6.2emGFP vector administered with Exgen 500 (Euromedex) reagent and glucose 5% (v/v)	Intratumoral inj.	20 μg	Single dose	Significant decrease of tumor growth and weight	Not assessed	Tréhoux et al., 2015
Hs766t-L2 initially transfected with a miR-29c agomir (200 nM)	miR-29c	RT	OX	nude mice	agomiR-29c	Intraperitoneal inj.	5 nmol	Twice weekly for 2 weeks	Reduced liver metastasis	Not assessed	Zou et al., 2015

**Table 5:** ***In vivo* studies in animal models of colorectal adenocarcinoma**.

**Xenografted cell line**	**MiRNA(s) of interest**	**Therapeutic strategy**	**Animal model**	**Animal strain (gender)**	**Delivery system and chemical modifications**	**Route of administration**	**Dosage**	**Frequency of administration**	**Results**	**Toxicity, undesired effects**	**Reference**
LS174T	miR-33a	RT	SX	Athymic nude mice (Hsd:Athymic Nude-Foxn1nu)	PEI complexes (PEI F25-LM/miRNA)	Intraperitoneal inj.	0.77 nmol (10 μg)	Three times per week for 25 days	Reduction in tumor proliferation and growth	No changes in body weight, behavioral alterations, or other signs of discomfort, no changes in ALT and AST, no induction of TNFα	Ibrahim et al., 2011
HCT-116	miR-145					intratumoral inj.	0.3 nmol (4 μg)				
HCT-116	miR-218	RT	SX	Nude mice (females)	miR-218 or control miR preincubated with Lipofectamine 2000 (Invitrogen)	Intratumoral inj.	1.2 nmol	Every 3 days	Inhibition of tumor growth	Not assessed	He et al., 2012
LoVo	miR-K-ras	RT	SX	SCID-C.B-17/IcrHsd-Prkdcscid mice (females)	Plasmid DNA encoding miRNA specific to K-ras	Intratumoral inj. followed by percutaneous electroporation	50 μg	Single dose	Transient suppression of tumor growth for 6 days, increased necrosis	No side effects observed	Vidic et al., 2010
MC38 (murine colon adenocarcinoma)	miR-27a	RT	SX	Normal C57B/l6 mice	miR-27a precursor (GenePharma) with Lipofectamine 2000 (Invitrogen)	Intratumoral inj.	6.26 μg	Every 3 days for 3 times	Inhibition of tumor growth, reduction of tumor sizes and weight	Not assessed	Bao et al., 2014
HCT-116	miR-133a	RT	SX	BALB/c nude mice (females)	miR-133a preincubated with Lipofectamine 2000 (Invitrogen)	Intratumoral inj.	0.3 nmol	Every 3 days for 4 times	Reduced tumor growth rate	Not assessed	Dong et al., 2013
HT29	let-7	IT + immunotherapy	SX	Athymic nude mice (females)	Anti-Fas activating mAb clone CH11 (Millipore Corporate)	Intratumoral inj.	20 μg	Three times (days 4, 6, 8)	Reduction in tumor size, increased sensitivity of tumor cells to Fas-related apoptosis	Not assessed	Geng et al., 2011
					let-7 inhibitor (GMR-miR™ microRNA inhibitor, GenePharma)		20 μg				
DLD1 (KRAS^G13D^)	miR-4689	RT	SX	Nude mice (females)	Carbonate apatite nanoparticles conjugated with mature hsa-miRNA (Gene Design)	Tail-vein inj.	40 μg	Three times a week for 8 times	Inhibition of tumor growth	No mortalities or body weight loss, no significant differences in blood chemistry tests, except for the slight increase in BUN, histological damage not observed (brain, heart, lung, liver, kidney, spleen, small intestine, and colon)	Hiraki et al., 2015
SW620	miR-429	RT	SX	Nude mice (males)	Mature miRNA	Intratumoral inj.	1 μg	Single dose	Inhibition of tumor growth, reduction of tumor weight	Not assessed	Sun Y. et al., 2014
SW620	miR-27b	RT	SX	NOD/SCID mice (females)	Cholesterol-conjugated mimics (GenePharma)	Intratumoral inj.	1 OD/mouse	Twice a week for 5 weeks	Inhibition of angiogenesis, severe tumor necrosis, one xenograft disappeared completely (a scab remained)	Two mice from negative control group died after 4-week treatment, cause of death was not determined	Ye et al., 2013

Toxicity assessment was part of 11 studies. It was performed at least as animal body weight control but usually was followed by animal behavior observation, histopathology examination of tissue dissections of various organs (brain, heart, liver, lungs, spleen, kidney), or blood biochemistry with regard to liver and kidney functions (blood urea nitrogen, liver enzymes, bilirubin). There were two declared deaths of experimental animals in the selected studies. These mice were administered cholesterol-conjugated oligonucleotide, but both were from the negative control group. The cause of death was not determined (Ye et al., 2013). One study declared slight but statistically significant elevation of blood urea nitrogen in the group of mice treated systemically with mature miRNA conjugated with carbonate apatite nanoparticles (Hiraki et al., 2015). Transient hepatotoxicity was found in mice systemically treated with adenoviral vector Ad-L5-8miR148aT, but the symptoms were milder than those produced by administration of Ad-wt (Bofill-De Ros et al., 2015). No immune response to the RNA-based treatment was reported in the selected studies, as they used immune deficient strains. Depending on the specific genotype, nude or severe immunodeficient (SCID) mice lack normal cytokine production together with other immune impairments.

The selected studies utilized various types of administered miRNA-based substances and different delivery systems. These issues and their fine tuning are the main points in the successful development of a miRNA-based therapy. Ability to overcome natural barriers that face transferring an oligonucleotide into the cell has to be balanced with the extent of toxicity, as systems with good cell penetration are usually more cytotoxic in a non-specific manner. To bring a complex sight on the development of miRNA-based therapy in gastrointestinal cancer, we gathered relevant information about the type of substances, delivery systems and routes of administration used in the selected 26 studies and we discuss them in detail. The issue of toxicity is described for each delivery system and later on also for the concept of miRNA-based therapy itself.

## Important issues in the field of miRNA-based therapy preclinical testing

### Routes of administration and delivery systems

MiRNA-based therapeutics in animal studies summarized in this review were administered either systemically, or locally. In systemic delivery, the intravenous (tail-vein) and intraperitoneal injections were used (Figure 6). Local administration was performed as intratumoral injection into the subcutaneous tumors. Delivery systems employed in the presented studies include viral vectors, biocompatible cationic polymers and copolymers, inorganic nanoparticles, atelocollagen, and liposomes.

#### Viral vectors

Viral vectors could be administered both locally, and systemically and they include lentiviruses, adenoviruses, and adeno-associated viruses (Chen et al., 2015). Viral delivery of antisense construct expression vectors was used to inhibit miR-21 and miR-148a in animal models of pancreatic cancer (Bao et al., 2014; Bofill-De Ros et al., 2015), while expression vector for miR-1266/1207-5p was examined in replacement therapy in gastric carcinoma (Chen et al., 2014). Viruses are able to effectively deliver miRNA therapeutics (precursors, mimics, genes, or inhibitors) into the tumor cell, but their use could be associated with the risk of insertional mutagenesis, gain of replication competency of viral particles, or immune activation. Nucleic acid of adenoviruses (dsDNA viruses) and adeno-associated viruses (ssDNA viruses) usually do not integrate into the host cell genome, while lentiviral (ssRNA viruses) integrates (Soriano et al., 2013; Chen et al., 2015). Adeno-associated viruses are generally less immunogenic, but adenovirus-based delivery system could produce at least transient hepatotoxicity (Broderick and Zamore, 2011; Aslam et al., 2012) as was also observed by Bofill-De Ros et al. in animal model of pancreatic ductal adenocarcinoma (Bofill-De Ros et al., 2015).

#### Cationic polymer polyethylenimine

In the selected studies, the most frequently used synthetic polymer was polyethylenimine (PEI). It was utilized to deliver mimics or expression vectors of miR-34a, miR-206, and miR-217 in animal model of pancreatic cancer, or miR-33a and miR-145 in the model of colorectal carcinoma (Tables 4, 5). PEI is cationic polymer able to produce nanoparticles. It has linear or branched structure and different molecular weight according to the reaction conditions during the synthesis. Due to the positive charge, PEI has high capacity for negatively charged oligonucleotides and nucleic acids which are moreover condensed after complexation with PEI, and thus protected from nucleases. The charge of PEI also facilitate cellular uptake by electrostatic interaction with negatively charged surface molecules (e.g., heparin sulfate proteoglycans), after which the particles enter the cell by endocytosis. PEI is able to disrupt the endosome and release the cargo into the cytoplasm, which grants this method high transfection efficacy. The disruption of endosome is achieved by protonization of PEI and buffering of acidic environment of the vesicle. These processes are followed by osmolarity changes and water intake which leads to the swelling and burst of the endosome (Höbel and Aigner, 2013; Zhang et al., 2013). Better capacity and efficacy is achieved by branched PEI but at the cost of higher non-specific cytotoxicity. In the presented studies, mostly linear PEI is used, like commercially available transfection reagents ExGen500™ (Euromedex, Mundolsheim, France) and *in vivo*-jetPEI™ (Polyplus Transfection, Illkirch, France) assigned for *in vivo* experiments. Other issues associated with PEI delivery are an aggregation of created nanoparticles, or opsonization in the plasma recognized by phagocytes. PEI with high density of positive charge could also trigger erythrocyte aggregation and thrombosis (Kanasty et al., 2012). PEI particles could be conjugated with various molecules [e.g., polyethylene glycol (PEG), or antibodies] to resolve such difficulties (Malek et al., 2009). PEI is not a biodegradable polymer, thus its toxicity is intensively discussed. It depends strongly on molecular weight and branching (Fischer et al., 1999) and also on the cargo, as it may neutralize the charge of PEI. There are studies describing immune activation *in vivo*, (Beyerle et al., 2011) hepatotoxicity and lethality in mice (Chollet et al., 2002) and increased apoptosis *in vitro* (Merkel et al., 2011), and also those that proved no immune response, or hepatotoxicity in mice (Bonnet et al., 2008).

#### Inorganic nanoparticles—iron oxide, and carbonate apatite

Sun et al. used iron oxide nanoparticles to deliver miR-16 and overcome doxorubicin resistance in animal model of gastric adenocarcinoma (Sun Z. et al., 2014). Iron oxide nanoparticles (IONPs) are biocompatible and biodegradable particles with magnetic properties. They are composed of magnetite [Fe_3_O_4_, iron (II,III) oxide], or maghemite (Fe_2_O_3_, ferric oxide) and are usually coated with various other molecules (PEI, PEG, chitosan etc.) to improve their properties (Kievit and Zhang, 2011). IONPs could also serve as theranostics (i.e., substances with both diagnostic and therapeutic purpose). As well as other nanoparticles, IONPs protect nucleic acids from being cleaved by nucleases (Kievit et al., 2009) but could also be opsonized in the plasma and recognized by phagocytes, mainly by the reticuloendothelial system (RES). Non-coated IONPs are distributed in heart, liver, spleen, lungs, kidney, brain, stomach, small intestine, and bone marrow, while the highest concentration are reached in the liver and spleen due to the elimination by RES and macrophages (Wang et al., 2010). IONPs enter the cell by endocytosis and are degraded in the endosomes (Xie et al., 2009). Particles between 10 and 60 nm are the most effective, as they undergo limited kidney and liver/RES uptake, and are absorbed by tumor cells (Kievit and Zhang, 2011). Cytotoxicity of IONPs coated with PEI occurs *in vitro* in higher concentration than is needed for sufficient transfection (Lellouche et al., 2015). The administration *in vivo* could increase blood iron and intracellularly increase oxidative stress (Mahmoudi et al., 2011). Non-coated particles could produce hepatotoxicity, and lung or kidney damage (Hanini et al., 2011).

Study of Hiraki et al. describes utilization of different inorganic nanomaterial, carbonate apatite nanoparticles. They used these particles as a delivery system for mature miR-4689 in animal model of colorectal adenocarcinoma (Hiraki et al., 2015). Carbonate apatite [Ca_10_(PO_4_)_6−X_(CO_3_)_X_(OH)_2_] is composed of calcium cations and phosphate and carbonate anions in defined ratios. It was firstly described as a transfection reagent and a delivery system for plasmid DNA by Chowdhury et al. (2006). Nanoparticles of carbonate apatite are stable in plasma (pH = 7.4), protect nucleic acids from nuclease cleavage, but in acidic environment of endosomes, they are quickly degraded. Their cargo is then released and could probably escape from endosomes, as high effectivity of this transfection method was proved for DNA (Wu et al., 2015) and RNA (Hossain et al., 2010). In mice, these nanoparticles are accumulated in tumor probably due to the EPR effect (discussed below), but slight accumulation was found also in the liver (Wu et al., 2015). As this method arose from calcium phosphate co-precipitation, which is known to produce certain level of cytotoxicity *in vitro*, adverse effects in animals were inquired. In mice, Wu et al. declared no mortality, weight loss, or histological damage in liver, kidney, and spleen after administration of common dose, and also after 2.5 and 5-fold higher doses. They also do not observed any urinary calculi in a mouse model of repeated administration. The team advanced to the evaluation of the delivery system on monkeys (macaques *Macaca fascicularis*, formerly *M. cynomolgus*). Monkeys received repeated i.v. infusions during a movement restraint. Equivocal results were obtained, as some animals had reversible increase in AST, ALT, LDH, and CPK enzymes, but from further analyses of isoenzymes, authors suggested that these increments might arise from the stress associated with body restriction rather from heart or liver damage (Wu et al., 2015).

#### Atelocollagen

For direct intratumoral treatment, atelocollagen was used in the study of Frampton et al. in pancreatic ductal adenocarcinoma to deliver miR-21, miR-23a, and miR-27a (Frampton et al., 2011). Atelocollagen is a biocompatible and biodegradable polymer. It was developed and tested *in vivo* for gene (plasmid) delivery with controlled release by Ochiya et al. (Ochiya et al., 1999; Hao et al., 2016). Atelocollagen is prepared from collagen extracted from bovine dermis. Natural collagen contains specific amino acid sequences on both C- and N-terminus (“telopeptides”), which are highly immunogenic. By digestion with pepsin, these telopeptides are cleaved. The polymer is liquid at low temperatures, but solidifies at temperatures above 30°C (Ochiya et al., 2001; Komatsu et al., 2016). After intramuscular injection of plasmid DNA in complex with glucose and atelocollagen in mice, the transfection was efficient and last more than 60 days. No apparent toxicity, or hematologic changes were observed in this study, (Ochiya et al., 1999) as well as Frampton et al. described neither changes in mice body weight, nor serious adverse effects (Frampton et al., 2011).

#### Cationic lipids and liposomes

For intratumoral administration, several studies used lipid-based transfection reagent Lipofectamine 2000 (Thermo Fisher Scientific, Waltham, USA) designed originally for *in vitro* experiments, e.g., Dong et al. in animal model of colorectal adenocarcinoma to deliver miR-133a (Dong et al., 2013). Pramanik et al. utilized DOTAP (*N*-[1-(2,3-dioleoyloxy)propyl]-*N,N,N*-trimethylammonium methyl-sulfate) with co-lipids formula to deliver plasmid expression vector of miR-34a, and miR-143/145 cluster systemically in animal model of pancreatic ductal adenocarcinoma (Pramanik et al., 2011). Both delivery systems are composed of cationic lipids that form liposomes, vesicles with lipophilic bilayer and aqueous core able to encapsulate hydrophilic molecules (Mallick and Choi, 2014). By electrostatic interaction, cationic lipids have increased capacity for negatively charged nucleic acids (Xue et al., 2015). They enter the cells by endocytosis and are able to destabilize and breach endosomal membrane by interaction with its phospholipids (Zelphati and Szoka, 1996). Cationic lipids exert detergent effect on lipid membranes and interact also with enzymes, thus could irritate cells, decrease proliferation, alter gene expression, and even trigger cell lysis (Wu et al., 2001). They share the same advantages and disadvantages that account for positive charge as cationic polymers. They form aggregates with plasma proteins leading to RES elimination and accumulation in spleen and liver (Nchinda et al., 2002; Zhang et al., 2012). With decrease of positive charge, RNA encapsulation and transfection efficacy is decreasing. PEGylation increases blood circulation time of liposomes, (Pathak et al., 2011; Suk et al., 2016) but could lead to the formation of anti-PEG IgM antibodies (Ishida et al., 2006). Liposomes are generally less immunogenic than cationic polymers. But after processing of liposomes, some RNA molecules might remain on the surface of a particle. In the studies using siRNA, these residues lead to the significant immune activation (Xue et al., 2015). Inflammatory response in the liver followed by hepatotoxicity and with higher doses even lethality was described (Tan and Huang, 2002; Zhang et al., 2005).

### Pharmacokinetics of therapeutic oligonucleotides

#### Chemistry, physico-chemical properties, and absorption

Pharmacokinetics of therapeutically administered oligonucleotides is strongly driven by their physico-chemical properties. Generally, these properties are not sequence-specific in qualitative point of view, but can be quantitatively different from sequence to sequence, and could differ also between chemistries. Native oligonucleotides are small, negatively charged molecules, which means that the transfer through lipophilic membranes necessary for the absorption into systemic blood circulation and also later into the intracellular space is quite problematic.

The most common change in oligonucleotide chemistry is the replacement of phosphodiester bond with phosphorothioate bond in the backbone. This change goes usually hand in hand with chemical modification of the 2′ functional group on ribose in the nucleotide (*2*′-hydroxyl could be substituted e.g., to *2*′*-O*-methyl, *2*′*-O*-methoxyethyl, or *2*′-fluoro group), and conjugation with cholesterol. These modifications either increase the stability of oligonucleotides modifying their susceptibility to RNAse cleavage (phosphorothioate bonds, *2*′*-O*-modifications), or increase cellular uptake of the molecule (cholesterol conjugation). Cholesterol-conjugated *2*′*-O*-methyl/methoxyethyl-modified oligonucleotides are sometimes termed “agomiRs” or “antagomiRs” depending on their mechanism of action, and they were utilized in some of the studies focused on gastrointestinal cancer presented in this article (Chang et al., 2013; Wang et al., 2013; Zhang et al., 2015; Zou et al., 2015). Modification on *2*′ position could also change the affinity of oligonucleotides to plasma proteins which has a high impact on pharmacokinetics, most importantly on distribution and excretion (Crooke, 2007).

Another possibility to change oligonucleotide structure is chemical modification of the ribose forming a *2*′*,4*′-bicyclic structure, which is termed locked nucleic acid (LNA) (Kumar et al., 1998). The most common type of LNA is oligonucleotide with one or more *2*′*-O-4*′-methylene-β*-D*-ribosyl structure. This bicyclic bridge locks ribose in one of its conformation increasing binding affinity and decrease the susceptibility to nuclease cleavage (Braasch and Corey, 2001).

Various chemical modifications in the oligonucleotide structure are now available owing to the development of commercially available miRNA mimics. According to the information provided by manufacturers, miRNA mimics should possess higher affinity to miRISC and thus to the mRNA of interest. MiRNA-mimics should have no off-target biological activities due to the passenger strand, and should exert higher effect than native mature miRNAs. The chemistry modifications differ between passenger and guide strand, and the molecules could also be triple-stranded (e.g., Exiqon, Vedbaek, Denmark). Detailed information about the specific chemistry of the miRNA mimic are usually not released. Some evidences were published last year, that bring the commercially available miRNA mimics into focus because of non-specific dampening effect on overall gene expression, accumulation of non-endogenous high molecular weight RNA species and unintentional passenger strand loading into the RISC discovered after transient transfection of human cell lines. Søkilde et al. describe variations even between batches of a commercially available miRNA mimic obtained from one manufacturer (Søkilde et al., 2015). The authors emphasize the issue of a proper dosage of miRNA mimic and its optimization, and suggest to prefer viral and genetic approaches, as the created transcripts follow the physiological biosynthesis pathway and their mechanism of action could be considered as the very same as endogenous miRNAs (Jin et al., 2015).

Undesirable physico-chemical properties of oligonucleotides could be attenuated by delivery systems mentioned before, which subsequently influence pharmacokinetic processes of miRNA-based therapeutics.

#### Distribution, protein binding, and tissue accumulation

After being absorbed or injected into systemic circulation, charged molecules of oligonucleotides bind to various plasma proteins, above all on albumin and α_2_-macroglobulin (Cheng et al., 2013). The binding and the distribution is non-linear, saturable, changes slightly with length and sequence of oligonucleotides and is different in rodents and in human. Distribution to the tissues is very quick and prevails over metabolic degradation (Levin, 1999). Naked oligonucleotides accumulate in the liver, kidneys, spleen, bone marrow and lymphatic nodes, while they do not cross the blood-brain barrier, placental barrier and they are not present in *testes*.

In the treatment of cancer, accumulation of a drug in the tumor tissue or in the metastasis site is a desirable state. MiRNA-based therapeutics could achieve this due to enhanced permeability and retention (EPR) effect of a tumor. Enhanced permeability of new vessels and relative lack of lymphatic vessels in the tumor site was firstly described by Matsumura and Maeda (1986). Charge-neutral small particles complexed or loaded with miRNA-based therapeutics have enhanced extravasation and could accumulate in the tumor. Metastatic sites are generally less accessible, as their EPR effect is not so significant (Maeda, 2015).

According to the technology of the delivery system used, miRNA-based therapeutic could accumulate also extratumorally in various tissues. All cells capable of phagocytosis accumulate naked oligonucleotides, liposomes, or nanoparticles, e.g., RES cells present in the liver (Kupfer cells) and in the circulation, tissue monocytes and macrophages, and proximal tubular cells (Chen et al., 2015). In this case, the delivery system alone as a protection could be insufficient, because in plasma, these particles get coated by proteins recognized by the RES. The most common defense against RES is PEGylation, binding of polyethylene glycol substituents on the surface of a nanoparticle or liposome, which prevent binding of opsonization proteins and became very common. Contrarily, the excess of PEG on the surface of a delivery system particle could diminish cellular uptake, therefore the process of PEGylation should be optimized (Seto, 2010).

Oligonucleotides, liposomes and polymer-based nanocarriers enter the cell by active mechanism, endocytosis. Escape from endosomes is desired to reach the interaction of miRNA with mRNA, however, this is another obstacle in miRNA-based therapy. Some of the carriers could enhance endosomal escape by steric or osmotic effects. pH sensitive molecules could change structure in relatively acidic environment due to electrostatic interactions, which is leading to the mechanical disruption of the vesicle and release of miRNA into cytoplasm (Ju et al., 2014). Other molecules are accepting H^+^ (proton sponges) and by alteration of ion homeostasis cause swelling and burst of the endosome (Akinc et al., 2005; Chen et al., 2015).

#### Metabolism of miRNA-based therapeutics

Ubiquitous nucleases begin to degrade oligonucleotides shortly after administration. According to the chemistry changes, free oligonucleotides are metabolized by 3′- and 5′-exonucleases or by endonucleases, and the rate of metabolism depends on the chemical modifications. Endonuclease cleavage is slower and takes place only when 3′ and 5′ end of oligonucleotide is protected by methoxyethyl-modified nucleotides. As was mentioned before, modifications on *2*′-hydroxyl on ribose or structural changes in the backbone such as LNA structure can decrease the affinity of nucleases to cleave miRNA-based therapeutics. Also the complexes of oligonucleotides with nanoparticles or liposomes have modified susceptibility to nuclease cleavage.

The metabolites of nuclease cleavage are weakly bound to the plasma proteins and therefore are rapidly excreted in urine. Oligonucleotides do not undergo liver oxidation by cytochrome P450, or conjugation processes (Levin, 1999; Crooke, 2007).

#### Excretion

Oligonucleotides not bound to proteins are excreted in the urine, while binding to plasma proteins, or other delivery systems like liposomes and nanoparticles of specific parameters (e.g., hydrodynamic diameter up to 5–6 nm) results in protection from being urinary excreted (Crooke, 2007; Cheng et al., 2013). As oligonucleotides accumulate also in the liver, they could be excreted by both these organs. About 10% of the administered dose of naked oligonucleotides, and 80% of the metabolites are urinary excreted. The remaining are excreted by *faeces*, or endure bound to the tissue, or inside the cells. The elimination half-life of oligonucleotides is 1–30 days depending on the type of tissue. This attribute allows designing a therapeutic regimen comfortable for potential patients with one dose administration for a week, 2 weeks, or a month (Crooke, 2007).

### Toxicity of miRNA-based therapy

The toxicity of oligonucleotide administration was largely studied in the field of antisense therapy. In miRNA-based therapy specifically, toxicity assessments are not a part every *in vivo* study and we have very limited information from the first phases of clinical research. For antisense oligonucleotides not targeted to miRNAs, there are evidences from rodent and non-rodent animal models, and also from human volunteers. Potential adverse effects could be provoked by hybridization-dependent or independent mechanisms, and could be linked with specific sequence motifs or length of an oligonucleotide. It means that some of the findings from antisense therapy in general are quite relevant for extrapolation to miRNA-based therapy.

Immunostimulation was described for phosphorothioate antisense oligonucleotides. It is a sequence-dependent, hybridization-independent process, which leads to the reversible activation of various immune cells (e.g., NK cells, B lymphocytes, mononuclear cells) and increased production of cytokines such as IL-6, IL-12 and interferon γ (Levin, 1999; Henry et al., 2002). The main responsible sequence motif is CpG (p stands for phosphodiester bond) or CG palindromic sequences naturally occurring mostly in bacterial genome (Krieg et al., 1995). While unmethylated, this motif is recognized by TLR receptors on immune cells and activates them. The effect is also exerted by oligonucleotides with both phosphorothioate, and phosphodiester bonds in the structure. In rodents, which are more sensitive than primates to this effect, splenomegaly, lymphoid hyperplasia, and multiple organ mononuclear infiltrates were described (Levin, 1999).

Another severe adverse effect relating with immunity is an activation of complement cascade. It prevails over TLR-mediated immune stimulation in primates and its mechanism is probably hybridization/sequence-independent originating from physico-chemical properties (polyanionic character) of oligonucleotides. In the study of Henry et al., after reaching a threshold plasma concentration after i.v. infusion, macaques suffered from emesis, ataxia, and facial edema. Hemodynamic changes (fluctuation of blood pressure and tachycardia), changes in blood count (neutropenia followed by neutrophilia), and increase of cytokines mentioned above were described (Henry et al., 2002). When maintaining plasma concentration below the threshold, symptoms were mild or not present, which is in accordance with the results of phase I clinical study with antisense oligonucleotide against intercellular adhesion molecule-1 (ICAM-1) (Glover et al., 1997).

Similar hybridization/sequence-independent mechanism leads also to the influencing of blood coagulation cascade observed in rodents, primates and human (Glover et al., 1997; Henry et al., 1997). Negative charge of oligonucleotides could inhibit intrinsic tenase complex (consisting of factor IXa and VIIIa, which activate factor X), and thus leads to the reversible prolonging of blood clotting and to the increase of activated partial thromboplastin time (aPTT) (Sheehan and Lan, 1998; Levin, 1999).

After administration of relatively high doses of antisense oligonucleotides (above 100 mg/kg in rodents), histological or laboratory signs of hepatotoxicity and renal toxicity were present in experimental animals. Mostly, immune-mediated cellular infiltrations in liver, multi-focal liver necrosis, and proximal tubules infiltrations were found in rodents. Posology studies indicate that lower doses (below 3 mg/kg) do not cause liver and kidney pathologies in monkey and human (Levin, 1999).

Different mechanism could potentially lead to hepatotoxicity, which was proven in rodents (Grimm et al., 2006). By introducing oligonucleotides into the cell, enzymes and other proteins that physiologically deal with these molecules could be saturated, and thus processing of other endogenous RNAs sharing these pathways could be diminished (Bader et al., 2010). This effect was described on mice treated with shRNA (short hairpin RNA) expression vectors. Mice suffered from multifocal liver necrosis followed by ascites, edema, increase of bilirubin and liver enzymes, and decrease of plasma proteins and body weight. Several mice died within 1 month. There were no signs of blood count changes, or increases in cytokine productions. ShRNAs compete of Dicer cleavage and exportin-5 stabilization in cytoplasm with endogenous pre-miRNAs, therefore mature liver miRNAs were found decreased and shRNAs precursors increased in mice with symptoms of hepatotoxicity. As Grimm et al. studied almost 50 distinct shRNAs, they assume that the effect was not sequence related (Grimm et al., 2006). Introducing of miRNA-precursors into the cell could produce the same effect, but the data concerning safety of miRNA-based therapies are limited. Again, proper posology studies are needed.

Another possible mechanism of toxicity is hybridization-dependent. But the toxicity arisen from both binding to the desired mRNAs, and off-target binding is hypothesized to be rare (Bader et al., 2010; van Rooij et al., 2012). MiRNA-mimics and precursors are suggested to be generally better tolerated than antisense therapy (Bader et al., 2010). One miRNA could regulate number of genes, frequently functionally linked in a specific pathway. Targeting more genes in one or more pathologically deregulated pathway could be beneficial. The potential for targeting other genes in different pathways still remains, but influencing of target or off-target genes with impact on cell viability should be revealed during accurate *in vitro* testing.

## MiRNA-based therapeutics in clinical trials

Certain chemical modifications of oligonucleotides structure and also several delivery systems for miRNAs have already entered clinical phase of drug development. There are no reports of clinical trials of miRNA-based therapies in gastrointestinal malignancies on which we have focused in this review—colorectal, pancreatic, gallbladder and gastric cancer. Two experimental miRNA-based therapies are now listed on ClinicalTrials.gov. MiR-34a mimics in an amphoteric liposomal formulation administered i.v. are tested in the phase I in patients with primary liver cancer and advanced or metastatic lung and kidney cancer, melanoma, multiple myeloma and lymphoma (NCT01829971, Adams et al., 2015).

MiR-16 mimic is evaluated in the treatment of malignant pleural mesothelioma also in the phase I (NCT02369198). The therapeutic system used is termed TargomiR and it is based on specific nanoscale delivery system—nonliving bacterial minicells (EnGeneIC Delivery Vehicle, EnGeneIC, New York, USA), and targeted to cancer cells by an anti-EGFR antibody, since EGFR is known to be overexpressed by certain types of cancer. Kao et al. even published some of the preliminary results achieved in the cohort of six patients with malignant pleural mesothelioma describing significant radiologic and metabolic responses indicated by PET-scan (Kao et al., 2015; Quinn et al., 2015).

In non-cancer diseases, the first miRNA-based drug in clinical settings was miravirsen (LNA miR-122 inhibitor) tested as a hepatitis C treatment. The drug entered clinical trials phase II (NCT02031133, NCT02508090, NCT02452814), but van den Ree has recently referred that the development of miravirsen had been ceased. A more potent miR-122 inhibitor conjugated with *N*-acetylgalactosamine entered phase II (RG-101, 2016; van der Ree et al., 2016).

Other delivery systems, used in the selected animal studies as carriers for miRNA-based therapeutics, are evaluated in clinical trials for non-miRNA treatment. Future results from these trials may serve also for the development of miRNA-based therapies, as we may obtain e.g., the information about the potential toxicity, or pharmacokinetic aspects of a specific delivery system regardless of its cargo.

Lentiviral vectors are mostly used to transfect cells that are subsequently injected into the patient, e.g., in the treatment of lymphoma (NCT02337985). Adenoviral vectors are evaluated in various solid cancers and are usually administered locally, intraperitoneally, or even intratumorally. They are tested in urinary bladder cancer (NCT00003167), ovarian (NCT00964756), breast (NCT01703754), prostate (NCT01931046), or pancreatic carcinoma (NCT02705196). Adeno-associated viruses are tested in non-cancer diseases to deliver genes for the experimental treatment of hemophilia B (coagulation factor IX; NCT01620801), lipoprotein lipase deficiency (NCT00891306), Pompe disease (α-glucosidase; NCT00976352), or genetic retinopathies (NCT01482195). In gastric cancer, AAV is used to transfect patient's dendritic cells, which are later mixed with his T lymphocytes to produce specific cytotoxic T lymphocytes injected i.v. back to the patient (NCT02496273). IONPs are investigated in various applications in biomedicine, above all in diagnostics and tissue imaging (e.g., NCT00147238, NCT01895829). Ferumoxyde, superparamagnetic iron oxide, has already been used in clinical practice in the United States for the treatment iron-deficiency anemia in patients with chronic kidney disease. Finally, PEI particles for delivering gene therapy are utilized in the clinical trials phase I and II of pancreatic ductal adenocarcinoma (NCT01274455), hepatocellular carcinoma (NCT00825474) and urinary bladder carcinoma (NCT00595088, NCT01274455, NCT00393809).

## Future perspectives

In addition to animal models and techniques described in this review, there are also novel and promising approaches to target miRNAs under development. Very intriguing strategy present small-molecule inhibitors that target specific miRNAs (SMIRs, e.g., diazobenzene inhibiting miR-21) that usually interfere with miRNA biogenesis and maturation (Wen et al., 2015). SMIRs constitutes a reasonable and evidence based strategy with strong potential and chance for success. The progress of screening techniques and computational stimulation may address bright future in this field. CRISPR/Cas 9 technology is another emerging technique to be used in miRNA targeting therapy. For instance, construction of sequence specific CRISPR/Cas9 based miRNA inhibitor was reported to downregulate miR-17-92 cluster and miR-21, two canonical oncogenic miRNAs in cancer (Ho et al., 2015; Narayanan et al., 2016). Since single miRNA has the potential of regulating thousand genes, long non-coding RNA (lncRNA) that is capable of binding multiple miRNAs could consequently impact the expression of thousands of genes. In light of this potentially fundamental biological role, all the lncRNAs that act as endogenous miRNA sponges presents another promising strategy to target miRNAs in cancer. Finally, it can also be envisioned that blocking production, transportation and release of exosome miRNAs may have beneficial effects in controlling cancer development, and this may be achieved by targeting other non-cancerous cells such as the inflammatory cells in the cancer microenvironment.

## Conclusions

MiRNA-based therapies as a new class of targeted therapy are heading toward from bench to the bedside. It is now generally accepted and many times proved that influencing pathologically changed intracellular levels of miRNAs change oncogenic phenotype of cancer cells *in vitro* and *in vivo*. However, as there is no ideal animal model of a human pathology, the translational potential of most studies is somehow limited. In the studies selected for this review, change of a specific miRNA was followed by significant diminishing of tumor size or volume *in vivo*. The subcutaneous tumor model used in the bulk of the studies clearly do not respond with microenvironment of the normal tumor cells, and also the necessary immunodeficiency of experimental animals do not correspond with immune status of an average oncology patient, nevertheless, the results of animal studies are promising.

Serious obstacles still lie in the way to the clinical practice. The main issue is efficient delivery of miRNA-mimics, precursors, expression vectors, or inhibitors. Other important difficulty is an assessment of a proper dose sufficient for anticipated intracellular effects, but lacking or possessing acceptable adverse effects relating to immunostimulation, blood coagulation, or toxicities that account for the specific delivery systems. We also see the importance of non-rodent models in the development of new drugs, as shown on the immunostimulation triggered by oligonucleotides which is significantly different in nature in rodents and primates.

Several miRNAs and delivery system are now tested in clinical trials. Most of them are in phase I or II. Together with more information obtained from preclinical experiments, the results could move us forward on the way to a new approach in targeted therapy—drugs that aim on epigenetic mechanisms of pathophysiological processes.

## Author contributions

JM did the literature search and wrote the text, JM and OS made the figures, RD and OS advised on the concept of the review and lately rearranged, corrected and critically revised the text.

## Funding

This work was supported by the grant GA16-18257S of The Grant Agency of Czech Republic, by the internal Masaryk University Faculty of Medicine grants MUNI/A/1284/2015 and MUNI/11/InGA09/2014, by the Ministry of Education, Youth and Sports of the Czech Republic under the project CEITEC 2020 (LQ1601) and by the Czech Ministry of Health under the project MZ CR – RVO (MOU, 00209805).

### Conflict of interest statement

The authors declare that the research was conducted in the absence of any commercial or financial relationships that could be construed as a potential conflict of interest.
